# Massively parallel nanowell-based single-cell gene expression profiling

**DOI:** 10.1186/s12864-017-3893-1

**Published:** 2017-07-07

**Authors:** Leonard D. Goldstein, Ying-Jiun Jasmine Chen, Jude Dunne, Alain Mir, Hermann Hubschle, Joseph Guillory, Wenlin Yuan, Jingli Zhang, Jeremy Stinson, Bijay Jaiswal, Kanika Bajaj Pahuja, Ishminder Mann, Thomas Schaal, Leo Chan, Sangeetha Anandakrishnan, Chun-wah Lin, Patricio Espinoza, Syed Husain, Harris Shapiro, Karthikeyan Swaminathan, Sherry Wei, Maithreyan Srinivasan, Somasekar Seshagiri, Zora Modrusan

**Affiliations:** 1 Molecular Biology Department, Genentech Inc., 1 DNA Way, South San Francisco, CA 94080 USA; 2 Wafergen Biosystems Inc., 34700 Campus Drive, Fremont, CA 94555 USA; 3 Axiocor Inc., St. Catharines, ON L2N 5P6 Canada

**Keywords:** Single cell profiling, RNA sequencing, Single-cell transcriptome

## Abstract

**Background:**

Technological advances have enabled transcriptome characterization of cell types at the single-cell level providing new biological insights. New methods that enable simple yet high-throughput single-cell expression profiling are highly desirable.

**Results:**

Here we report a novel nanowell-based single-cell RNA sequencing system, ICELL8, which enables processing of thousands of cells per sample. The system employs a 5,184-nanowell-containing microchip to capture ~1,300 single cells and process them. Each nanowell contains preprinted oligonucleotides encoding poly-d(T), a unique well barcode, and a unique molecular identifier. The ICELL8 system uses imaging software to identify nanowells containing viable single cells and only wells with single cells are processed into sequencing libraries. Here, we report the performance and utility of ICELL8 using samples of increasing complexity from cultured cells to mouse solid tissue samples. Our assessment of the system to discriminate between mixed human and mouse cells showed that ICELL8 has a low cell multiplet rate (< 3%) and low cross-cell contamination. We characterized single-cell transcriptomes of more than a thousand cultured human and mouse cells as well as 468 mouse pancreatic islets cells. We were able to identify distinct cell types in pancreatic islets, including alpha, beta, delta and gamma cells.

**Conclusions:**

Overall, ICELL8 provides efficient and cost-effective single-cell expression profiling of thousands of cells, allowing researchers to decipher single-cell transcriptomes within complex biological samples.

**Electronic supplementary material:**

The online version of this article (doi:10.1186/s12864-017-3893-1) contains supplementary material, which is available to authorized users.

## Background

Single-cell RNA sequencing (RNA-seq) has rapidly evolved over the last few years, providing new understanding of cell composition and identity in normal and diseased settings [1, 2]. Transcriptional profiling at the single-cell level facilitates identification of new cell types and understanding of cellular heterogeneity, aids in lineage tracing, and elucidates hierarchical relationships among cell types in the course of development or disease progression [2–7]. For example, based on their transcriptome patterns, more than 40 subtypes of neurons were identified in the mouse cortex [7, 8]. Single-cell RNA-seq also identified a rare cell type in colon tissue [4].

Initial transcriptional profiling at the single-cell level was done using fluorescence-activated cell sorting (FACS). However, FACS-based techniques have limited throughput and are not cost-effective. Recently, methods that enable single-cell transcriptome and whole-genome sequencing without the need for FACS have been reported. Notably, microfluidic-based single-cell isolation (e.g. Fluidigm C1) and transcriptome analysis was performed successfully, though the throughput and the ability to capture a large range of cell sizes is limiting [6]. The C1-based method does not employ molecular barcodes for eliminating PCR duplicates during cDNA conversion and can potentially lead to data bias [6, 9]. Recently, encapsulation of thousands of cells using droplet-based microfluidic methods for single-cell RNA-seq have been demonstrated [10, 11]. While droplet-based technology is encouraging, further development of the experimental technique and analysis tools are needed for its wider adoption [9, 12, 13].

**Fig. 1:**
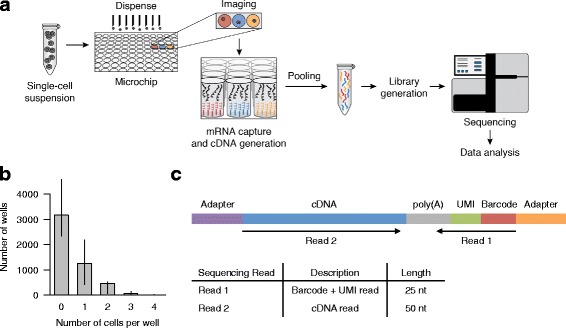
Overview of the ICELL8 single-cell RNA-seq workflow. **a** High-throughput single-cell RNA-seq is performed by dispensing a single-cell suspension onto a microchip (containing 5184 nanowells), followed by microchip imaging, and on-chip cDNA generation. The process is completed by standard in-tube NGS library generation, Illumina sequencing and computational data analysis. **b** Histogram of the number of wells containing 0, 1, 2, 3 or 4 cells as determined by image analysis software. Boxes and error bars indicated the median and the range, respectively, for several microchips (*n* = 5). **c** Schematic illustration of sequencing library constructs and sequence read position and length

Nanowell-based deposition of single cells is another promising approach for characterizing single-cell transcriptomes [14]. In this study we assessed the performance and utility of the ICELL8 system for massively parallel single-cell gene expression profiling (Fig. 1a). The ICELL8 system uses a multi-sample nanodispenser (MSND) to dispense single cells into a 5184-nanowell microchip. Imaging software is used to select single-cell-containing wells that are then processed to obtain single-cell transcriptome data. We showed that the system has a low rate of cell multiplets and high single-cell purity. In addition, we characterized transcriptomes of more than a thousand cultured cells and were able to identify representative cell subtypes from a complex tissue sample.

## Results

### Microchip for single-cell isolation

The ICELL8 microchip is made of aluminum alloy (41 mm^2^) and contains 5184 nanowells arranged in a square layout (72 × 72 wells). Each nanowell holds 150 nl and contains preprinted oligonucleotides; each oligonucleotide includes an oligo-(dT_30_) primer, a well-specific sequence (11 bp) used for cell barcoding and a unique molecular identifier (UMI, 10 bp; Fig. 1). The cell barcode is used to identify cDNA molecules generated from an individual cell, while UMIs identify individual mRNA molecules [15]. A similar microchip-based technology has been used previously in targeted sequencing applications [16, 17].

Cell suspensions were fluorescently labeled with live/dead stain (Hoechst 33324/Propidium Iodide, see Methods) prior to their dispensing into the microchip nanowells using the MSND (Additional file 1: Figure S1). The MSND is an 8-channel microsolenoid controlled dispenser that delivers ≥30 nl volumes using non-contact dispensing. The MSND dispenses up to eight different samples (one sample per channel) into a single microchip in approximately 12 minutes. To minimize evaporation, the microchip is enclosed in a controlled humidity and temperature chamber. Cross contamination between nanowells due to dispense tip misalignment was assessed using a checkerboard assay (see Methods, Additional file 2: Figure S2). In a test involving 11 MSND instruments we observed the average percentage of wells affected by misalignment to be 0.08%. Cells are dispensed by a limiting dilution; assuming a Poisson distribution for the number of cells per well, about one third of the 5184 nanowells contain a single cell under optimal conditions.

Following cell dispensing, the microchip was centrifuged to collect cells in a single plane and then imaged using a standard microscope with an automatic stage, a 4× objective and a charge-coupled device (CCD) camera. After imaging, the microchip was sealed and stored at −80 °C until ready for use (Additional file 1: Figure S1). Next we used imaging software (CellSelect, see Materials and Methods) to automatically and/or manually identify wells that contain single cells (Fig. 1b). A file containing positional information on identified candidate wells (dispense file) was then used to selectively deliver reverse transcriptase (RT) master mix to designated wells.

### Single-cell barcoding and sequencing

Cells in the microchip were lysed by freeze-thaw (see Methods). Under the directions from the dispense file, the MSND dispensed RT master mix into selected wells and cDNA synthesis was performed in those wells using the Single Cell Barcoding and Sequencing method (SCRB-seq) [18]. cDNAs from hundreds of cells were then pooled into a single tube, purified and amplified by standard practices. The amplified cDNAs were subjected to transposon-mediated fragmentation (“tagmentation”), PCR amplified and converted to an Illumina-compatible NGS library (Additional file 1: Figure S1). RNA-seq libraries were sequenced using paired-end sequencing where read 1 (25 bp) contained the well barcode and UMI and read 2 (50 bp) captured the cDNA sequence (Fig. 1c).

### Sequencing data processing and quality control

**Fig. 2:**
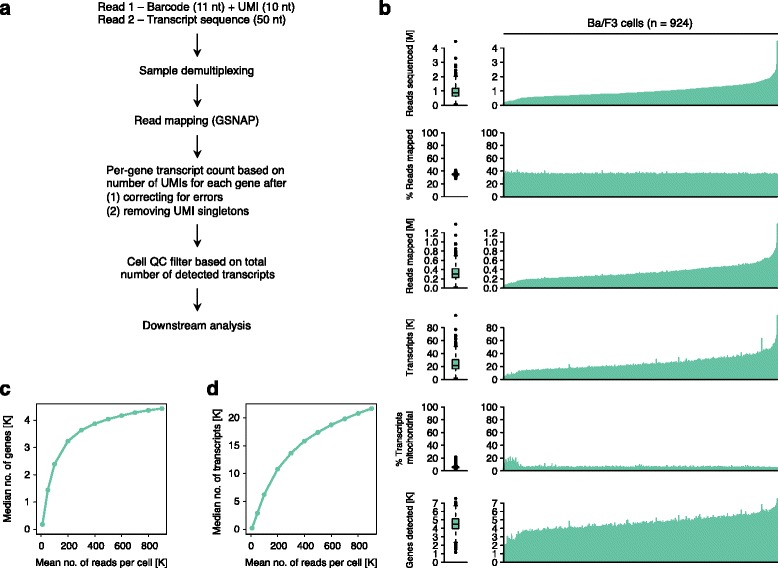
Sequence data from nanowell-based single-cell expression profiling. **a** Data analysis workflow. QC, quality control. **b** Quality control statistics for 924 Ba/F3 cells processed on one microchip, including total number of sequenced reads, alignment rate, number of mapped reads, total number of detected transcripts, percentage of transcripts corresponding to mitochondrial genes, and number of detected genes. Box plots indicate the interquartile range (IQR), *horizontal lines* are the median, whiskers extend to the most extreme data point no more than 1.5 x IQR from the box. **c** Median number of detected genes per cell for different sequencing depths. **d** Median number of detected transcripts per cell for different sequencing depths

To analyze sequencing reads generated from pooled single-cell RNA-seq libraries we developed a computational pipeline shown in Fig. 2a. Reads from different wells were demultiplexed based on a perfect match to the expected barcode sequences. After mapping to a reference genome, per-gene transcript counts were inferred based on the number of unique UMIs for each gene, after correcting for errors in the UMI sequence and excluding singleton UMIs represented by a single read only (see Methods). To assess data quality for a single-cell sequencing project, we inspected per-cell statistics as illustrated in Fig. 2b for 924 mouse Ba/F3 cells. Analyzed statistics included (1) total number of sequenced reads, (2) alignment rate, (3) number of mapped reads, (4) total number of transcripts, (5) percentage of transcripts corresponding to mitochondrial genes, and (6) number of detected genes. The percentage of unfiltered reads that could be mapped uniquely to the reference genome appeared constant across wells (~35%; Fig. 2b and Additional file 3: Figure S3a). We observed that the number of sequenced reads varied between nanowells (median 862,220; inter-quartile range 643,100–1,197,000) and increased linearly with the estimated number of captured transcripts (*r* = 0.99; Additional file 3: Figure S3b). This indicated that variation across nanowells was likely due to differences in cellular mRNA content or mRNA capture efficiency rather than variation in library construction or sequencing efficiency. Spatial plots of the number of detected transcripts per well did not reveal systematic effects due to well position (Additional file 4: Figure S4). Previous studies reported that mitochondrial reads may indicate poor-quality cells [19]. The percentage of mitochondrial transcripts was typically low (~6%); although we observed a higher percentage for some solid tissue samples (data not shown). We noticed that cells with poor quality data, having few detected genes and high mitochondrial content, were typically associated with low inferred transcript counts. We therefore defined a quality control criterion for processed cells by requiring a minimum number of detected transcripts for each cell. Appropriate cutoffs were determined separately for each data set (Additional file 5: Figure S5).

### Gene detection and reproducibility of single-cell expression profiles

To assess the sensitivity of the platform and allow comparison with other single-cell systems, we down-sampled reads for each cell and determined the median number of detected genes and transcripts with increasing sequencing depth (Fig. 2c and d). At an average sequencing depth of 100 K reads per cell we detected a median of ~2500 genes in Ba/F3 cells. The number of detected genes increased to >4000 at higher sequencing depths (Fig. 2c).

To assess the reproducibility between cells when profiling a relatively homogeneous sample type, we compared per-gene transcript counts between pairs of Ba/F3 cells. Pairwise comparisons generally showed high correlation with median *r* = 0.83 (Pearson correlation coefficient) and interquartile range of 0.81–0.84; an example is shown in Additional file 6: Figure S6a (*r* = 0.77). When performing the same analysis using per-gene read counts, we observed lower correlation (*r* = 0.69; Additional file 6: Figure S6b), illustrating the advantage of UMIs in reducing PCR amplification bias. Next we asked whether single-cell gene expression data accurately reflected expression profiles obtained from bulk cells. We processed total RNA from bulk Ba/F3 cells on the same microchip as Ba/F3 single cells and found that the bulk expression profile was highly correlated with the ensemble (average) of single-cell profiles (*r* = 0.95; Additional file 6: Figure S6c).

### Assessment of cell multiplet rate and single-cell impurity

**Fig. 3:**
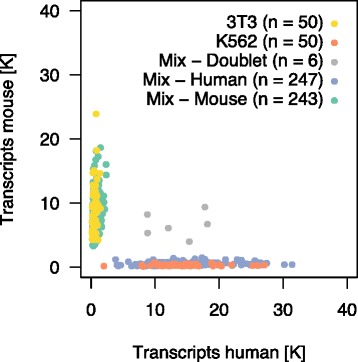
Species-mixing experiment. Single-cell expression data from one-to-one cell mixture of human K562 and mouse 3T3 cells, together with single-cell data from cell suspensions of 3T3 cells and K562 cells alone. Data were mapped independently to the human and mouse genome. Reads mapping to both genomes with ≤3 mismatches were excluded. For the one-to-one mixture, 247 cells and 243 cells were classified as human and mouse, respectively, 6 cells were classified as cross-species cell multiplets

An important determinant of the utility of a single-cell profiling platform is its ability to accurately partition individual cells, such that sequencing reads for each barcode are truly derived from a single cell [20]. Possible issues include multiple cells tagged with the same barcode (referred to as cell multiplets), and cross-contamination due to PCR chimera and/or free RNA from lysed cells (referred to as single-cell impurity). To assess these factors we performed a mixed-species experiment where a one-to-one mixture of human K562 cells and mouse 3T3 cells (*n* = 499), as well as K562 cells alone (*n* = 50) and 3T3 cells alone (*n* = 50), were processed on the same microchip (Fig. 3). We mapped the data independently to the human and mouse genome and excluded ambiguous reads that mapped to both genomes with ≤3 mismatches. The majority of cells from the one-to-one mixture displayed strong enrichment for transcripts specific to either one of the two species and were classified as human (*n* = 247) or mouse (*n* = 243; see Methods). Six cells (1.2%) had a high percentage of transcripts from both species and thus were classified as cross-species multiplets. Since the experiment only allowed us to identify mixed-species multiplets, possible multiplets consisting of cells from the same species remained undetected. Assuming that same-species multiplets occurred at a similar rate as cross-species multiplets, we estimated the overall multiplet rate as ~2.4%. The low cell multiplet rate indicated that the imaging software performed as expected and selected mostly single cells. We also observed that cells classified as human or mouse had on average 97% and 94% of transcripts corresponding to human and mouse, respectively, indicating high single-cell purity.

### Single-cell expression profiles of cultured cell lines

**Fig. 4:**
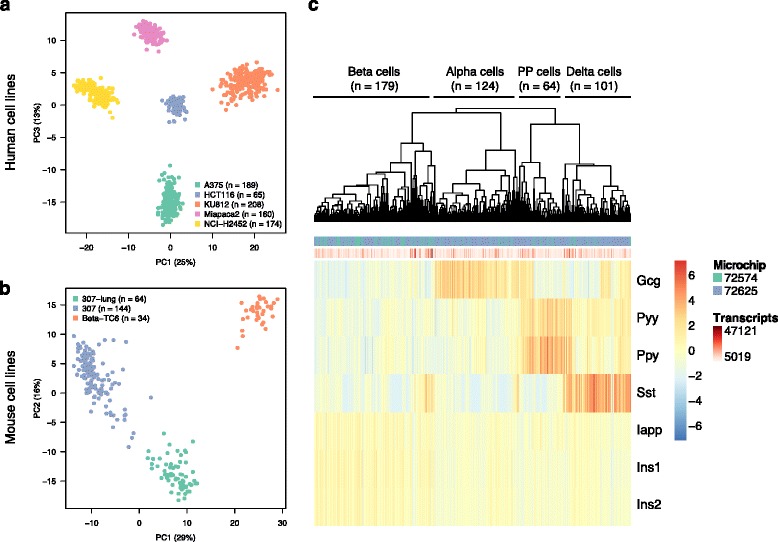
Single-cell RNA-seq profiling of cultured human and mouse cell lines and mouse pancreatic islets. **a** Unsupervised principal component analysis (PCA) for human cell lines based on the 500 most variable genes. **b** Unsupervised PCA for mouse cell lines based on the 500 most variable genes. **c** Hierarchical clustering of mouse pancreatic islet cells based on known cell type markers. Four subpopulations were identified based on four clusters indicated in the hierarchical clustering dendrogram. Cell type labels (alpha, beta, delta and PP) were assigned based on the expression of known marker genes

We next asked if the ICELL8 system is capable of distinguishing cultured cells derived from different tissue sources. We separately dispensed eight cell suspensions of five human (A375, HCT116, NCI-H2452, Miapaca2 and KU812) and three mouse (Beta-TC6, 307 and 307-lung) cell lines across two microchips, obtaining a total of 796 human and 242 mouse cells. Principal component analyses based on the 500 most variable genes, as well as hierarchical clustering based on the 100 most variable genes, showed clear separation of different cell lines (Fig. 4a and b, Additional file 7: Figure S7). Interestingly, mouse 307 and 307-lung cells showed more intra-cluster variability likely due to the fact that these cells were derived from tumors and have undergone minimal culturing, compared to the human cell lines which have been passaged for many generations. The most variable genes included many known markers of the cell line tissues of origin. These include hemoglobin genes (HBB, HBG1, HBG2) in the peripheral blood-derived cell line KU812, MIA (melanoma inhibitory activity) in the melanoma-derived cell line A375 [21] as well as insulin (Ins1, Ins2) and islet amyloid polypeptide (Iapp) in the mouse pancreatic beta cell line Beta-TC6 (Additional file 7: Figure S7).

### Identification of cell subtypes from pancreatic islets

Finally we determined whether the platform can distinguish cell types within a solid tissue sample. For this purpose we profiled 468 cells from adult mouse pancreatic islets. Pancreatic islets consist of the endocrine cells of the pancreas, with insulin-producing beta cells forming the majority. The other three major cell types include glucagon (Gcg)-producing alpha cells, somatostatin (Sst)-producing delta cells and pancreatic polypeptide (Ppy)-producing gamma cells (also known as PP cells). Hierarchical clustering based on known marker genes revealed the four distinct cell populations (Fig. 4c) and the relative abundance of cell types tracked the known composition of adult mouse pancreatic islets, including 38% beta cells (*n* = 179), 26% alpha cells (*n* = 124), 22% delta cells (*n* = 101), and 14% PP cells (*n* = 64). Unsupervised principal component analysis based on the 500 most variable genes did not show clear clusters of four major islet cell subtypes; however beta and alpha cells were largely separated (Additional file 8: Figure S8).

## Discussion

Transcriptome profiling of individual cells by single-cell RNA-seq is a powerful approach for deciphering the cell composition of complex tissues; however, broader use of this method has been limited by its ease of use, scalability, cost and turn-around time. Here we report a novel system for high-throughput single-cell RNA-seq, ICELL8, which uses nanowell-based cell capture and relies on cell- and transcript-specific barcoding to obtain transcript counts for individual cells. Although the ICELL8 system uses similar chemistry for single-cell RNA-seq library generation as other platforms, it features unique components, including i) an MSND instrument for dispensing single-cell suspensions into nanowells, ii) software for automated identification of hundreds to over a thousand of single-cell-containing wells, and iii) UMI-based transcript counting. The ease of dispensing cells of any size or shape into nanowells with the MSND eliminates errors associated with manual pipetting of viscous reagents into microfluidic devices. Cells as large as cardiomyocytes (~100 μm) and spheroids have been dispensed, imaged and processed for single-cell RNA-seq using the system (data not shown). The system also provides flexibility, allowing for processing of up to eight samples on the same microchip. Taken together, the ICELL8 system enables processing of several chips per day, which translates to processing tens of thousands of individual cells. Ease of use and fast turn-around time are key features of the system, which allow experiments with a variety of biological samples. Overall, it takes ~3 days on ICELL8 to process one sample from cell dispensing to library QC (see Additional file 1: Figure S1), compared to ~2 days on the 10× Genomics system. The consumable cost including chips, cell-handling reagents, enzymes and primers is in a range similar to many current commercial technologies.

Here we assessed the performance of the ICELL8 system by profiling a mixture of human and mouse cell lines to determine single-cell purity (94–97%) and cell multiplet rate (<3%). Performance characteristics were comparable to other single-cell RNA-seq platforms (e.g. multiplet rate in 10× Genomics: ~1% at 1000 cell density and ~6% at 6000 cell density [22]). In this study we profiled panels of human and mouse cell lines, as well as mouse pancreatic islets. Unsupervised principal component analysis did not show clear clusters of the four major islet cell subtypes [23]. Future versions of the ICELL8 system may benefit from improvements in sample processing including minimizing ambient RNA.

The ICELL8 system adds to the growing number of technologies that have been developed to understand biology at the single-cell level and complements existing microfluidic and droplet-based technologies. Besides enabling applications such as single-cell RNA-seq, it can empower other high-throughput sequencing applications that require imaging and selection to process only cells of interest. The current configuration uses nanowells with volumes of 150 nl. These wells can be configured to larger dimensions for volumes as high as 1 μl. Thus the ICELL8 system is capable of accommodating applications that require several reagent additions such as whole-genome amplification, combined readouts of DNA and RNA, or RNA and protein. Future technology improvements, including miniaturization that could result in a chip with >100 K nanowells, would enable enormously parallel processing of single cells. In addition, we have developed technologies where single cells can be targeted to occupy each well, thereby generating occupancy rates of >80% (http://www.freepatentsonline.com/20160045884.pdf). Furthermore, imaging enables the selection of wells not only based on Hoechst/Propidium Iodide staining but also based on fluorescent antibody staining. Thus, cells sorted by FACS can be orthogonally validated and selected and processed for single-cell RNA-seq and other applications.

## Conclusions

We demonstrated the performance and utility of a novel nanowell-based single-cell RNA sequencing system, ICELL8, which enables expression profiling of thousands of cells. Based on single-cell expression profiling data generated by ICELL8, we were able to identify representative cell types in mouse pancreatic islets and discriminate between mixed human and mouse cells. The technological advancements of ICELL8 enable more time- and cost-efficient transcriptome characterization of single cells, providing researchers with deeper insight into complex biological samples.

## Methods

### Single-cell samples

Human (K562, A375, HCT116, NCI-H2452, Miapaca2 and KU812) and mouse (3T3, Ba/F3, Beta-TC6, 307 and 307-lung) cell lines were used for evaluating the ICELL8 single-cell RNA-seq system. The melanoma cell line A375 was cultured in DMEM media with 10% fetal bovine serum (FBS, Thermo Fisher); the colon cancer cell line HCT116, mesothelioma cell line NCI-H2452, pancreatic adenocarcinoma cell line Miapaca2, chronic myelogenous leukemia (CML) cell line KU812 and K562 were cultured in RPMI 1640 media supplemented with 10% FBS. The mouse pancreatic beta cell line Beta-TC6 was cultured in DMEM media supplemented with 15% FBS; the mouse fibroblast 3 T3 cell line was cultured in DMEM supplemented with 10% FBS and 1 mM sodium pyruvate; the mouse pro-B cell line Ba/F3 was maintained in RPMI 1640 supplemented with 10% FBS and 2 ng/ml mouse IL-3 (R&D Systems). 307 and 307-lung cells were derived from a conditional Pik3ca knock-in mouse model [24] and were cultured in Epicult B basal medium (STEMCELL Technologies). All cell media contain 2 mM L-glutamine (except for 307 and 307-lung), 100 U/ml penicillin, and 100 mg/ml streptomycin. Cells were prepared as single-cell suspensions either by trypsinization and gentle washing, for adhesive cells, or direct washing, for suspension cells. Adult mouse pancreas was first perfused with the Liberase solution through the common bile duct, and then dissected and incubated at 37 °C for 10 min. Islets were separated from acinar tissue using histopaque density centrifugation (Sigma). Liberase solution was made by dissolving 5 mg Liberase TL (Roche) in 20 ml 1xHBSS buffer containing 25 mM HEPES (pH 7.2), 1.8 mM CaCl_2_, 10 μg/ml DNase and 0.1% BSA. Purified islets were dissociated into single cell suspension using Accumax cell dissociation solution (Innovative Cell Technologies) for up to 30 min at room temperature.

### Cell isolation by limiting dilution

Cells were stained with Hoechst 33324 and Propidium Iodide (Thermo Fisher) for 20 min. The cell viability and density was checked with ViCell XR (Beckman Coulter) using Trypan Blue. Cells were diluted to achieve a density of 1-2 cells per 50 nl in a final dispensing mix which contained a diluent, RNAsin (New England Biolab) and 0.35X PBS (without Ca^++^ and Mg^++^, pH 7.4, Thermo Fisher). A 384-well source plate with 8 designated wells containing cell suspensions, 1 well for positive control, 1 well for negative control, and 1 well for fiducial mix (fluorescent dye permitting image alignment confirmation) was placed in the MSND (WaferGen). Each of the 8 sample source wells in the 384-source plate was sampled by 1 of the 8 channels in MSND. Cells, positive controls, negative controls, and fiducial mix, were dispensed onto one chip within 16 min. Each well received 50 nl of either cell mix, positive control, negative control, or fiducial mix. Total RNA (~10 pg) from K562 cells was dispensed into selected nanowells and used as in-process positive controls. For Ba/F3 data, total RNA (~10 pg) from Ba/F3 cells was dispensed into selected nanowells and used as bulk in analyses.

### MSND quality testing

For each tested MSND, cross contamination between nanowells was measured using a checkerboard assay performed on a single microchip (Additional file 2: Figure S2). First negative control master mix (no template DNA) was dispensed into wells located on one half of the microchip (NTC wells, *n* = 2520). The percentage of wells that showed signal was considered background unrelated to misalignment of the dispensing tips. In the second half of the chip, lambda DNA master mix (Positive wells, *n* = 1024) and negative control master mix (Test wells, *n* = 1496) were dispensed in an alternate checkerboard pattern. The dispensed chip was sealed with a film, centrifuged and a real-time PCR analysis was performed. If the dispenser tip is misaligned, lambda DNA master mix is inadvertently added to test wells, resulting in Ct values indicative of cross contamination. Ct values were used to determine the number of Test wells and NTC wells that showed signal. To ensure that only intended PCR products were quantified, a melt-curve analysis was performed. Test wells and NTC wells with melting temperature 3 standard deviations outside of the mean melting temperature for Positive wells were excluded. Percentage of misalignment was calculated as the difference between the percentage of Test wells with signal minus the percentage of NTC wells with signal (i.e. % misalignment = % Test wells that show signal - % NTC wells that show signal).

### Microchip imaging and selection of single-cell-containing nanowells

After dispensing, each chip was sealed and centrifuged at 300 g for 5 min at 4 °C before imaging with a Leica microscope (Leica). A total of 288 images, 144 each for Hoechst 33,342 and for Propidium Iodide were captured. Each image comprised the picture of 36 wells. Following imaging (~7 min), the microchip was stored at −80 °C for at least 45 min or until ready for further processing.

Microchip images were analyzed using CellSelect software (WaferGen) to determine the viability and number of cells present in each nanowell. Briefly, to identify cells in an image, a Laplacian of Gaussian (LoG) image was calculated with a user-selectable scale. The LoG image was then segmented using auto-provided or user-defined thresholds. The resulting shapes were then classified by user-defined size and shape. Objects that do not meet the criteria were rejected. These steps eliminate many artifacts that may appear in the microscope images. This segmentation and classification process was performed for images obtained using both Hoechst 33324 (channel 1) and Propidium Iodide (PI, channel 2) images. In the default configuration, cells visible in channel 2 indicate PI positive (dead cells) which are auto-excluded from further processing. Using the default configuration, CellSelect identified all nanowells that have one cell in channel 1 (Hoechst) and no cells in channel 2 (PI). Nanowells that had one bright cell and additional dim cells or debris were further excluded. In summary, nanowells that contained only one cell were selected as candidates and additional visual inspection was performed to confirm the presence of single viable cells. After selecting the highest confidence candidate cells, the software auto-generated multiple files used to streamline the downstream processing (e.g. sample-barcode file, filter file for dispensing reverse transcription mix, cell dispense and Poisson summary statistics).

### Single-cell cDNA generation

Microchips were removed from −80 °C and left at room temperature for 10 min. Cells were lysed by freeze-thaw at this step. Chips were then centrifuged at 3800 g for 5 min at 4 °C and transferred to a thermocycler with a program of 72 °C for 3 min and 4 °C hold to anneal preprinted oligonucleotides to polyA mRNAs. The microchips were centrifuged as previously and were placed back into the MSND. A separate 384-well source plate containing reverse transcription (RT) reagents (1 mM dNTP, 1 μM E5 Oligos, Maxima H Minus RT buffer and 12 U/μl of Maxima H Minus reverse transcriptase) in 4 wells was used in the MSND, which delivered 50 nl of reverse transcription mix to selected nanowells. The microchips were spun down and transferred to a thermocycler with a program of 42 °C for 90 min and 4 °C hold to perform RT. Post reaction chips were inverted and centrifuged (3800 g 10 min at 4 °C) to simultaneously collect and pool well contents into a single microcentrifuge collection tube. Double-stranded cDNA was cleaned by the DNA Clean & Concentrator™-5 kit (Zymo Research). Eluate was treated with Exonuclease I (37 °C for 30 min, 80 °C for 20 min). A PCR program of 95 °C for 1 min, 18 cycles of 95 °C for 15 s, 65 °C for 30 s, 68 °C for 6 min, and 1 cycle of 72 °C for 10 min and 4 °C hold was performed using Advantage 2 polymerase. Amplicons were purified using Agencourt AMPure XP magnetic beads (Beckman Coulter). cDNA quality was assessed using a Bioanalyzer High Sensitivity DNA chip (Agilent Technologies) and quantity was determined by a Qubit High Sensitivity kit (Thermo Fisher Scientific).

### RNA-seq library construction and sequencing

One ng of cDNA was used for library construction using the Nextera XT kit (Illumina) according to manufacturer’s instruction. A custom-made Nextera P5 (WaferGen) and a P7 index primer provided by the Nextera XT kit (Illumina) were used to amplify the “tagmented” fragments. Libraries were purified and size selected using Agencourt AMPure XP magnetic beads (Beckman Coulter) to obtain an average library size of 500 bp. A typical yield for a library comprised of ~1500 cells was ~15 nM. Libraries were sequenced on either a HiSeq2500 or HiSeq4000 (Illumina) to obtain on average ~ 1-2 million 25 × 50 bp reads per cell.

### RNA-seq data processing

Read pairs were de-multiplexed requiring a perfect match between the first 11 bases of read 1 and one of the expected barcodes. The second read was then mapped to the reference genome using GSNAP [25] retaining the UMI sequence for each aligned read. Only uniquely mapping reads were considered. After mapping, the number of captured transcripts per gene was inferred based on UMIs. Read pairs with UMIs containing Ns were excluded. To avoid inflation of transcript counts due to errors in UMI sequences, we clustered UMIs with similar sequences for each gene (allowing for 1 mismatch). To remove low-abundance UMIs that may be the result of chimeric PCR products (Additional file 9: Figure S9), we only considered UMI clusters represented by at least two reads. Per-gene transcript counts were based on the number of distinct UMI clusters. For downstream analyses, per-gene transcript counts were normalized by dividing the counts for each cell by a cell-specific scale factor, calculated as the total transcript count for a given cell, divided by the median total transcript count across cells. No additional normalization was performed between microchips.

### Read depth simulation

We assessed the effect of sequencing depth on the number of detected genes and transcripts using Ba/F3 data. Ba/F3 cells were sequenced at an average read depth D of ~1 M reads per cell. To simulate an average read depth d < D, for each cell we sampled a fraction d/D from the subset of reads aligning to annotated genes, and recalculated the number of detected genes and transcripts as described above.

### Species-mixing experiment

Sequence reads were mapped independently to the human and mouse genomes. Reads that mapped to both genomes with ≤3 mismatches were excluded. In Fig. 3 the total number of detected mouse transcripts was plotted against the total number of detected human transcripts for each cell. Cells that had more than 2925 human transcripts were classified as human (*n* = 247). Cells that had more than 1955 mouse transcripts were classified as mouse (*n* = 243). Cells that were classified as both human and mouse were considered cross-species multiplets (*n* = 6). Cells that were not classified as either species were excluded from the analysis (*n* = 3). The cutoff for human (mouse) cells was determined by considering cells with more transcripts mapping to mouse (human) and computing the median plus five times the inter-quartile range of the number of human (mouse) transcripts. Single-cell purity estimates for human (mouse) cells were obtained by considering cells classified as human (mouse), dividing the number of transcripts detected in human (mouse) by the sum of transcripts detected in either species, and taking the median across cells.

### Clustering analysis

Most variable genes were determined based on the variance of normalized transcript counts after log_2_(*x* + 1) transformation. PCA and hierarchical clustering was performed based on normalized transcript counts after log_2_(*x* + 1) transformation and mean-centering genes. Hierarchical clustering was performed with 1 - Pearson correlation as distance metric and average linkage using the R package NMF.

## Additional files

Additional file 1: Figure S1. Overview of single-cell RNA-seq workflow on the ICELL8 system with detailed information on each processing step, including the time required for completion. (PDF 80 kb)

Additional file 2: Figure S2. Checkerboard assay. (a) Image of a microchip where the right half contains negative control master mix (NTC wells, *n* = 2520) and the left half contains lambda DNA master mix master (Positive wells, *n* = 1024) and negative control master mix (Test wells, *n* = 1496) in a checkerboard pattern. (b) Number of Test wells with signal, number of NTC wells with signal, and calculated misalignment rate for 11 MSNDs and 19 microchips. (PDF 1288 kb)

Additional file 3: Figure S3. Well-to-well variation in the number of reads and detected transcripts for Ba/F3 cells. (a) Number of mapped reads plotted against number of sequenced reads for each well. (b) Number of detected transcripts plotted against number of sequenced reads for each well. (PDF 237 kb)

Additional file 4: Figure S4. Heatmaps illustrating the total number of detected transcripts for each well selected for downstream processing. Data are for three microchips, each with 5184 wells arranged in a 72 × 72 square layout. Microchips 72,618 and 72,598 were used for profiling human and mouse cell lines (names of cell lines indicated in the plot). Microchip 72,625 was used for profiling pancreatic islets. For microchips with multiple dispensed samples, the dispense area for each sample is indicated. (PDF 93 kb)

Additional file 5: Figure S5. Percentage of mitochondrial transcripts plotted against total number of detected transcripts for mouse Ba/F3 cells (a), human cell lines (b), mouse cell lines (c), and pancreatic islets (d). Dashed lines indicate the minimum number of detected transcripts required as a cell QC filter for each data set. (PDF 409 kb)

Additional file 6: Figure S6. Cell-to-cell variability and comparison of single-cell ensemble versus bulk expression. (a) Scatter plot of per-gene transcript counts for two Ba/F3 cells. (b) Scatter plot of per-gene read counts for two Ba/F3 cells shown in (a). (c) Scatter plot of per-gene transcript counts for bulk cells versus ensemble of single cells. (PDF 272 kb)

Additional file 7: Figure S7. Hierarchical clustering of (a) human and (b) mouse cell lines based on 100 most variable genes. (PDF 1272 kb)

Additional file 8: Figure S8. Unsupervised principal component analysis (PCA) for mouse pancreatic islet cells based on the 500 most variable genes. Circles correspond to cells and are colored by (a) microchip (*n* = 116 for 72574, *n* = 352 for 72625) and (b) transcript counts of cell type markers (Ins1, Gcg, Ppy, and Sst). (PDF 139 kb)

Additional file 9: Figure S9. Effect of requiring a minimum number of reads per UMI on single-cell purity. Shown are data for the species-mixing experiment when considering UMIs supported by a minimum of 1, 2 or 3 reads. Single-cell purity estimates are shown in each panel. Otherwise as Fig. 3. (PDF 94 kb)

## References

[1] Jaitin DA, Kenigsberg E, Keren-Shaul H, Elefant N, Paul F, Zaretsky I, Mildner A, Cohen N, Jung S, Tanay A (2014). Massively parallel single-cell RNA-seq for marker-free decomposition of tissues into cell types. Science.

[2] Patel AP, Tirosh I, Trombetta JJ, Shalek AK, Gillespie SM, Wakimoto H, Cahill DP, Nahed BV, Curry WT, Martuza RL (2014). Single-cell RNA-seq highlights intratumoral heterogeneity in primary glioblastoma. Science.

[3] Darmanis S, Sloan SA, Zhang Y, Enge M, Caneda C, Shuer LM, Hayden Gephart MG, Barres BA, Quake SR (2015). A survey of human brain transcriptome diversity at the single cell level. Proc Natl Acad Sci U S A.

[4] Grün D, Lyubimova A, Kester L, Wiebrands K, Basak O, Sasaki N, Clevers H, van Oudenaarden A (2015). Single-cell messenger RNA sequencing reveals rare intestinal cell types. Nature.

[5] Kim K-T, Lee HW, Lee H-O, Kim SC, Seo YJ, Chung W, Eum HH, Nam D-H, Kim J, Joo KM (2015). Single-cell mRNA sequencing identifies subclonal heterogeneity in anti-cancer drug responses of lung adenocarcinoma cells. Genome Biol.

[6] Treutlein B, Brownfield DG, Wu AR, Neff NF, Mantalas GL, Espinoza FH, Desai TJ, Krasnow MA, Quake SR (2014). Reconstructing lineage hierarchies of the distal lung epithelium using single-cell RNA-seq. Nature.

[7] Zeisel A, Muñoz-Manchado AB, Codeluppi S, Lönnerberg P, La Manno G, Juréus A, Marques S, Munguba H, He L, Betsholtz C (2015). Brain structure. Cell types in the mouse cortex and hippocampus revealed by single-cell RNA-seq. Science.

[8] Tasic B, Menon V, Nguyen TN, Kim TK, Jarsky T, Yao Z, Levi B, Gray LT, Sorensen SA, Dolbeare T (2016). Adult mouse cortical cell taxonomy revealed by single cell transcriptomics. Nat Neurosci.

[9] Grün D, van Oudenaarden A (2015). Design and analysis of single-cell sequencing experiments. Cell.

[10] Klein AM, Mazutis L, Akartuna I, Tallapragada N, Veres A, Li V, Peshkin L, Weitz DA, Kirschner MW (2015). Droplet barcoding for single-cell transcriptomics applied to embryonic stem cells. Cell.

[11] Macosko EZ, Basu A, Satija R, Nemesh J, Shekhar K, Goldman M, Tirosh I, Bialas AR, Kamitaki N, Martersteck EM (2015). Highly parallel genome-wide expression profiling of individual cells using Nanoliter droplets. Cell.

[12] Brennecke P, Anders S, Kim JK, Kołodziejczyk AA, Zhang X, Proserpio V, Baying B, Benes V, Teichmann SA, Marioni JC (2013). Accounting for technical noise in single-cell RNA-seq experiments. Nat Methods.

[13] Kharchenko PV, Silberstein L, Scadden DT (2014). Bayesian approach to single-cell differential expression analysis. Nat Methods.

[14] Fan HC, Fu GK, Fodor SPA (2015). Expression profiling Combinatorial labeling of single cells for gene expression cytometry Science.

[15] Islam S, Zeisel A, Joost S, La Manno G, Zajac P, Kasper M, Lönnerberg P, Linnarsson S (2014). Quantitative single-cell RNA-seq with unique molecular identifiers. Nat Methods.

[16] Herazo-Maya JD, Noth I, Duncan SR, Kim S, Ma S-F, Tseng GC, Feingold E, Juan-Guardela BM, Richards TJ, Lussier Y (2013). Peripheral blood mononuclear cell gene expression profiles predict poor outcome in idiopathic pulmonary fibrosis. Sci Transl Med.

[17] De Wilde B, Lefever S, Dong W, Dunne J, Husain S, Derveaux S, Hellemans J, Vandesompele J (2014). Target enrichment using parallel nanoliter quantitative PCR amplification. BMC Genomics.

[18] Soumillon M, Cacchiarelli D, Semrau S, van Oudenaarden A, Mikkelsen TS. Characterization of directed differentiation by high-throughput single-cell RNA-Seq. BioRxiv. 2014.

[19] Ilicic T, Kim JK, Kolodziejczyk AA, Bagger FO, McCarthy DJ, Marioni JC, et al. Classification of low quality cells from single-cell RNA-seq data. Genome Biol. 2016;17:29–43. 10.1186/s13059-016-0888-1 PMC4758103 26887813

[20] Xin Y, Kim J, Ni M, Wei Y, Okamoto H, Lee J, Adler C, Cavino K, Murphy AJ, Yancopoulos GD (2016). Use of the Fluidigm C1 platform for RNA sequencing of single mouse pancreatic islet cells. Proc Natl Acad Sci U S A.

[21] Riechers A, Bosserhoff AK (2014). Melanoma inhibitory activity in melanoma diagnostics and therapy - a small protein is looming large. Exp Dermatol.

[22] Zheng GXY, Terry JM, Belgrader P, Ryvkin P, Bent ZW, Wilson R, Ziraldo SB, Wheeler TD, McDermott GP, Zhu J, et al. Massively parallel digital transcriptional profiling of single cells. Nat Commun. 2017;8:14049–60. 10.1038/ncomms14049 PMC5241818 28091601

[23] Baron M, Veres A, Wolock SL, Faust AL, Gaujoux R, Vetere A, et al. A single-cell Transcriptomic map of the human and mouse pancreas reveals inter- and intra-cell population structure. Cell Systems. 2017;8:14049–60. 10.1016/j.cels.2016.08.011 PMC5228327 27667365

[24] Yuan W, Stawiski E, Janakiraman V, Chan E, Durinck S, Edgar KA, Kljavin NM, Rivers CS, Gnad F, Roose-Girma M (2013). Conditional activation of Pik3ca(H1047R) in a knock-in mouse model promotes mammary tumorigenesis and emergence of mutations. Oncogene.

[25] Wu TD, Nacu S (2010). Fast and SNP-tolerant detection of complex variants and splicing in short reads. Bioinformatics.

