# Supplementary figures and images for: Massively parallel nanowell-based single-cell gene expression profiling
Source: BMC Genomics. 2017 Jul 7;18:519. doi: 10.1186/s12864-017-3893-1 (PMC5501953; doi:10.1186/s12864-017-3893-1)

Fig. S1

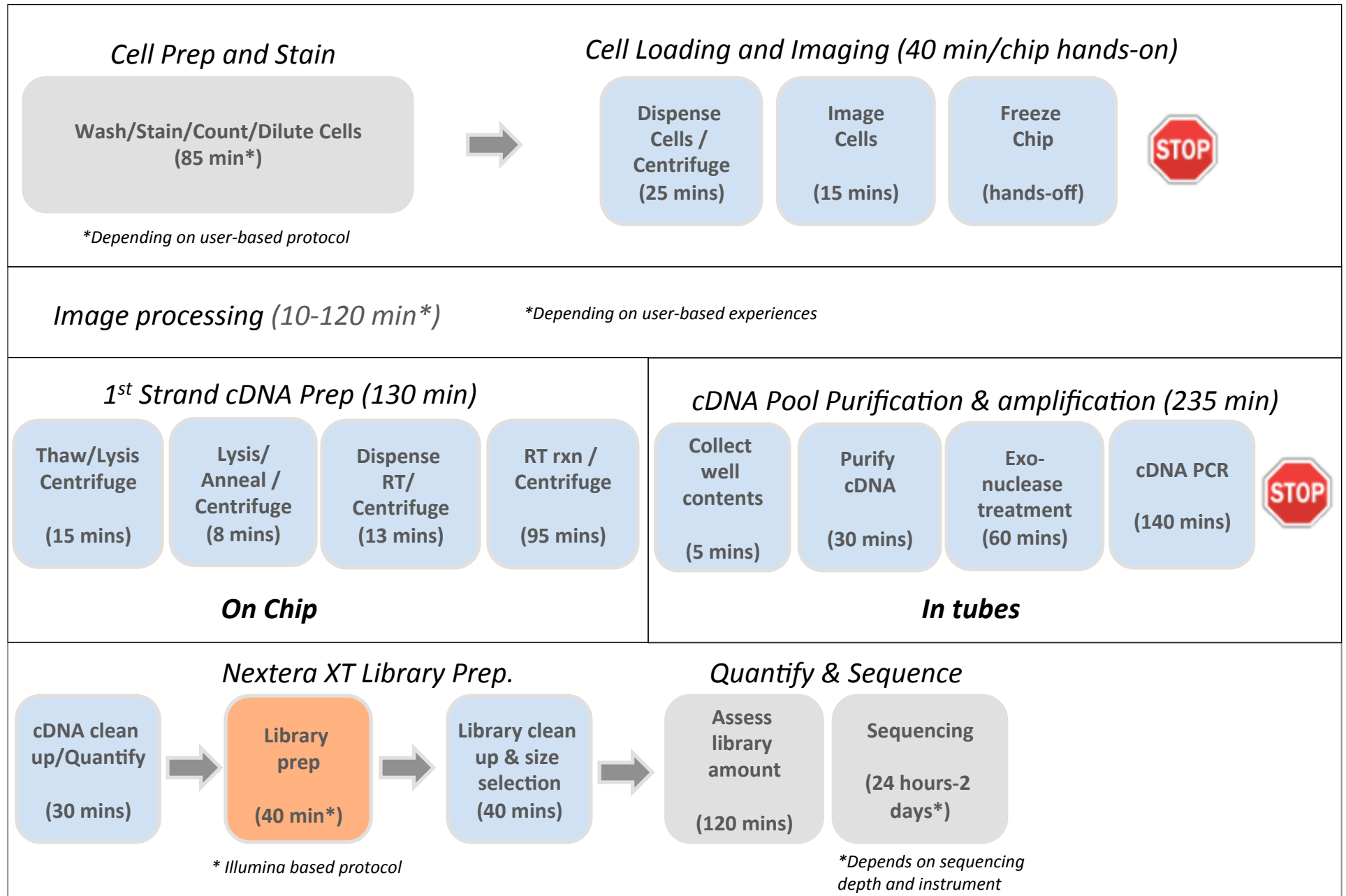

Supplement: Supplementary file 1 — Overview of single-cell RNA-seq workflow on the ICELL8 system with detailed information on each processing step, including the time required for completion. (PDF 80 kb) [file 12864_2017_3893_MOESM1_ESM.pdf]

Fig. S2

**a**

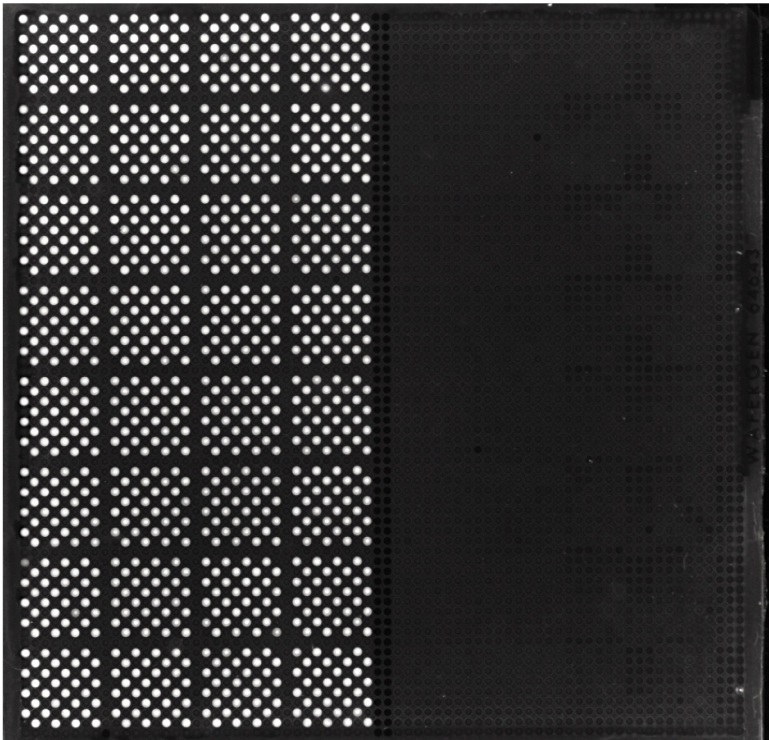

**b**

| MSND Number | Chip ID | Test wells (n = 1496) | NTC wells (n = 2520) | % Misalignment |
|-------------|---------|-----------------------|----------------------|----------------|
| 103         | 91040   | 3                     | 0                    | 0.20%          |
| 102         | 86911   | 0                     | 0                    | 0.00%          |
| 102         | 86937   | 2                     | 0                    | 0.13%          |
| 22          | 89015   | 2                     | 0                    | 0.13%          |
| 91          | 88696   | 4                     | 4                    | 0.11%          |
| 91          | 87769   | 1                     | 1                    | 0.03%          |
| 112         | 89029   | 0                     | 0                    | 0.00%          |
| 112         | 89048   | 1                     | 0                    | 0.07%          |
| 113         | 91801   | 3                     | 1                    | 0.16%          |
| 113         | 91811   | 0                     | 1                    | 0.00%          |
| 110         | 89008   | 1                     | 0                    | 0.07%          |
| 110         | 89003   | 2                     | 0                    | 0.13%          |
| 106         | 91802   | 1                     | 0                    | 0.07%          |
| 106         | 91791   | 4                     | 1                    | 0.23%          |
| 111         | 91812   | 1                     | 0                    | 0.07%          |
| 111         | 91808   | 0                     | 0                    | 0.00%          |
| 107         | 86935   | 1                     | 0                    | 0.07%          |
| 99          | 93517   | 0                     | 0                    | 0.00%          |
| 99          | 91035   | 0                     | 0                    | 0.00%          |
|             |         |                       |                      | <b>0.08%</b>   |

Supplement: Supplementary file 2 — Checkerboard assay. (a) Image of a microchip where the right half contains negative control master mix (NTC wells, n = 2520) and the left half contains lambda DNA master mix master (Positive wells, n = 1024) and negative control master mix (Test wells, n = 1496) in a checkerboard pattern. (b) Number of Test wells with signal, number of NTC wells with signal, and calculated misalignment rate for 11 MSNDs and 19 microchips. (PDF 1288 kb) [file 12864_2017_3893_MOESM2_ESM.pdf]

Fig. S3

**a**

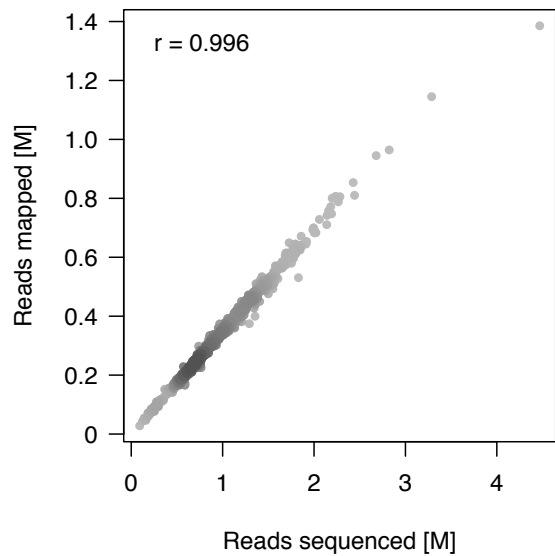

**b**

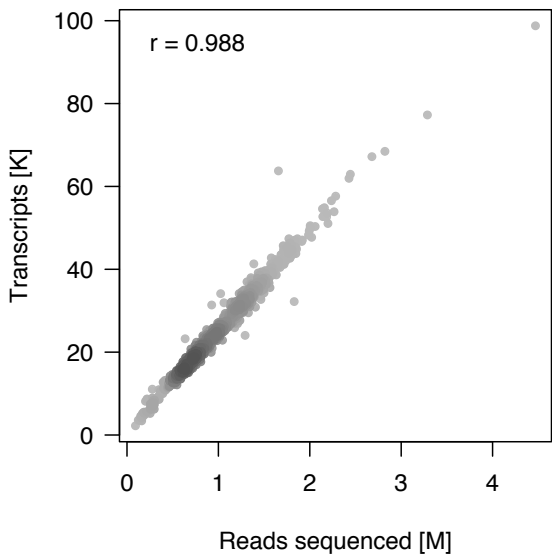

Supplement: Supplementary file 3 — Well-to-well variation in the number of reads and detected transcripts for Ba/F3 cells. (a) Number of mapped reads plotted against number of sequenced reads for each well. (b) Number of detected transcripts plotted against number of sequenced reads for each well. (PDF 237 kb) [file 12864_2017_3893_MOESM3_ESM.pdf]

Fig. S4

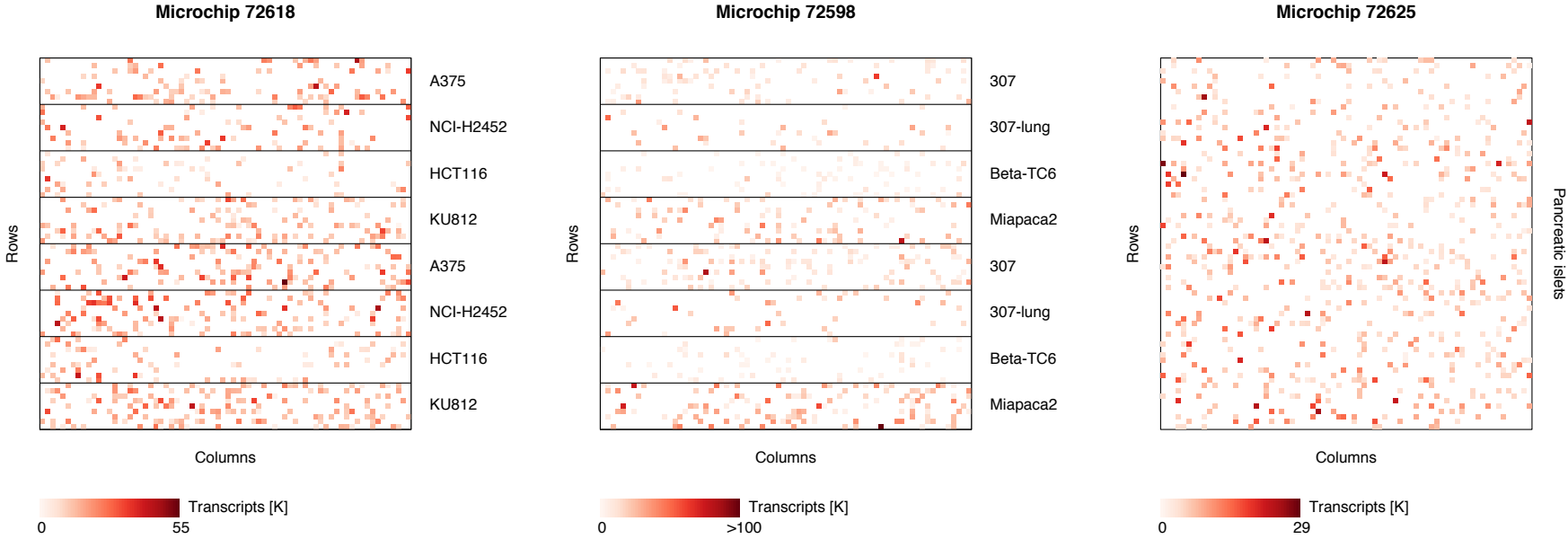

Supplement: Supplementary file 4 — Heatmaps illustrating the total number of detected transcripts for each well selected for downstream processing. Data are for three microchips, each with 5184 wells arranged in a 72 × 72 square layout. Microchips 72,618 and 72,598 were used for profiling human and mouse cell lines (names of cell lines indicated in the plot). Microchip 72,625 was used for profiling pancreatic islets. For microchips with multiple dispensed samples, the dispense area for each sample is indicated. (PDF 93 kb) [file 12864_2017_3893_MOESM4_ESM.pdf]

Fig. S5

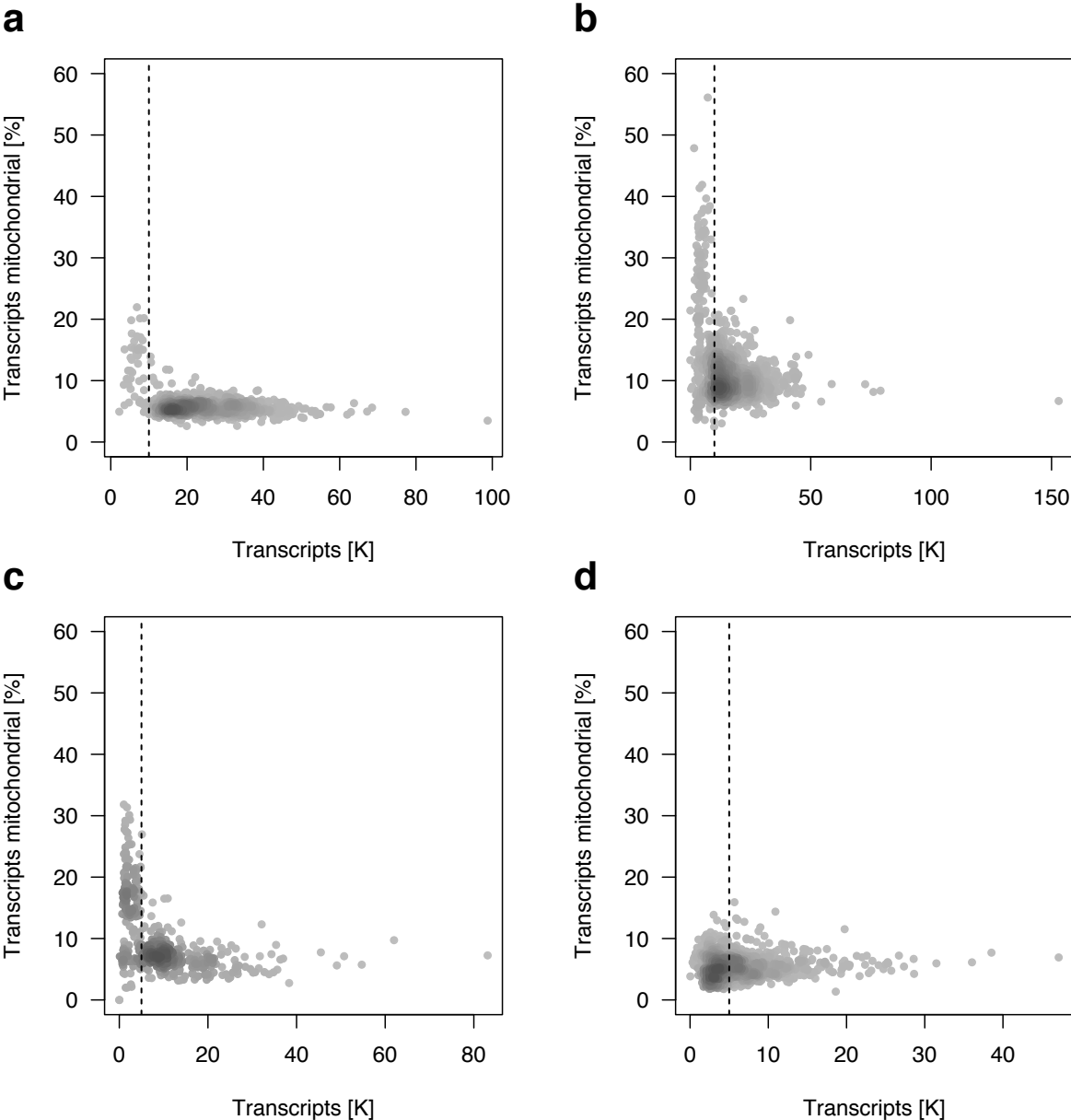

Supplement: Supplementary file 5 — Percentage of mitochondrial transcripts plotted against total number of detected transcripts for mouse Ba/F3 cells (a), human cell lines (b), mouse cell lines (c), and pancreatic islets (d). Dashed lines indicate the minimum number of detected transcripts required as a cell QC filter for each data set. (PDF 409 kb) [file 12864_2017_3893_MOESM5_ESM.pdf]

Fig. S6

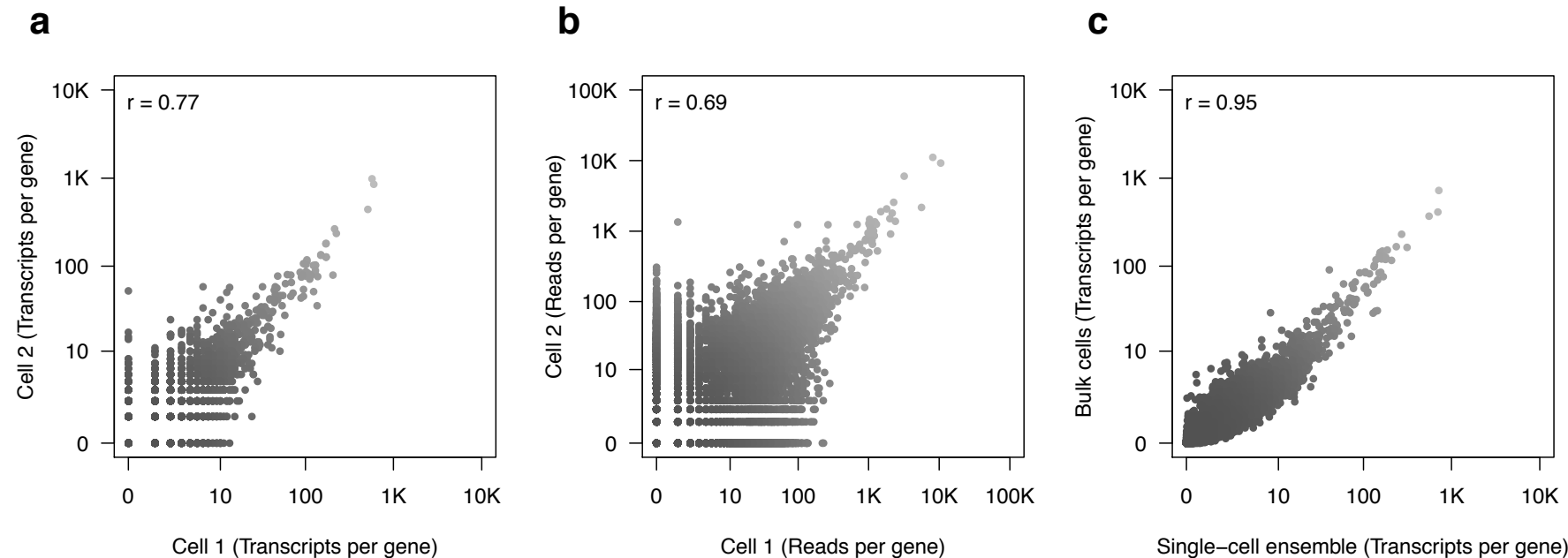

Supplement: Supplementary file 6 — Cell-to-cell variability and comparison of single-cell ensemble versus bulk expression. (a) Scatter plot of per-gene transcript counts for two Ba/F3 cells. (b) Scatter plot of per-gene read counts for two Ba/F3 cells shown in (a). (c) Scatter plot of per-gene transcript counts for bulk cells versus ensemble of single cells. (PDF 272 kb) [file 12864_2017_3893_MOESM6_ESM.pdf]

Fig. S7

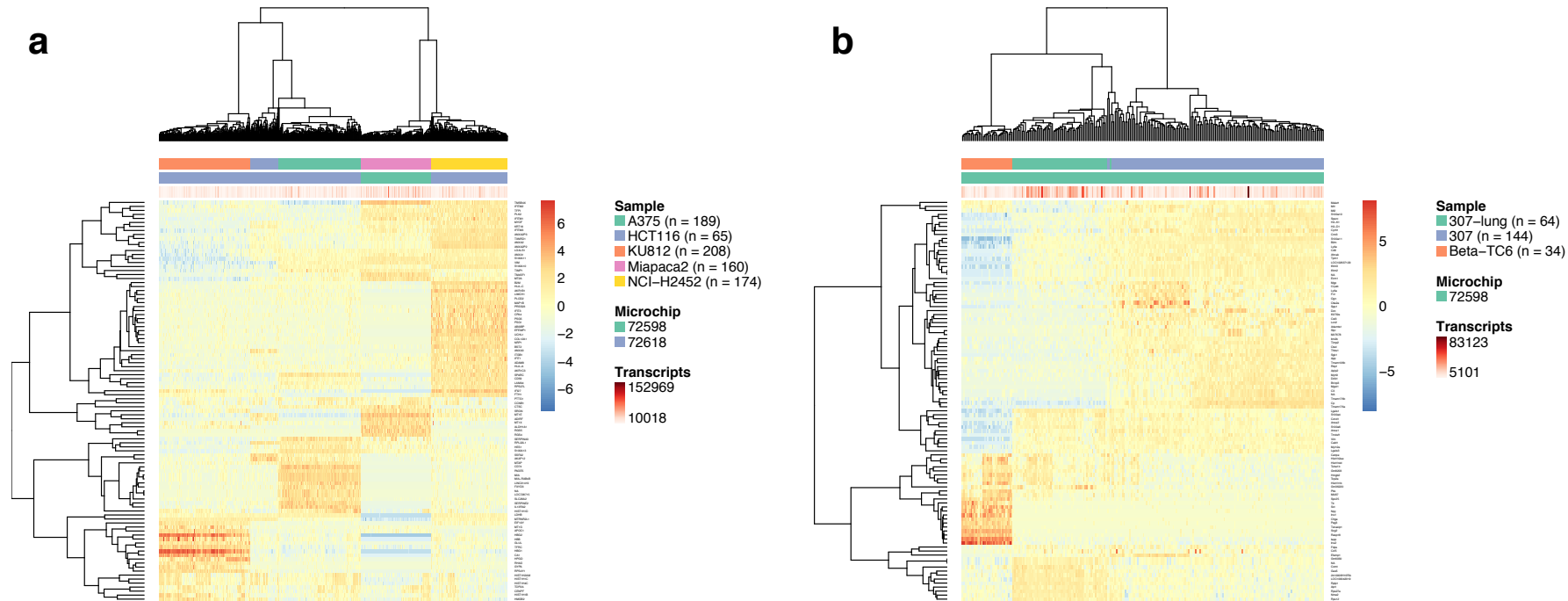

Supplement: Supplementary file 7 — Hierarchical clustering of (a) human and (b) mouse cell lines based on 100 most variable genes. (PDF 1272 kb) [file 12864_2017_3893_MOESM7_ESM.pdf]

Fig. S8

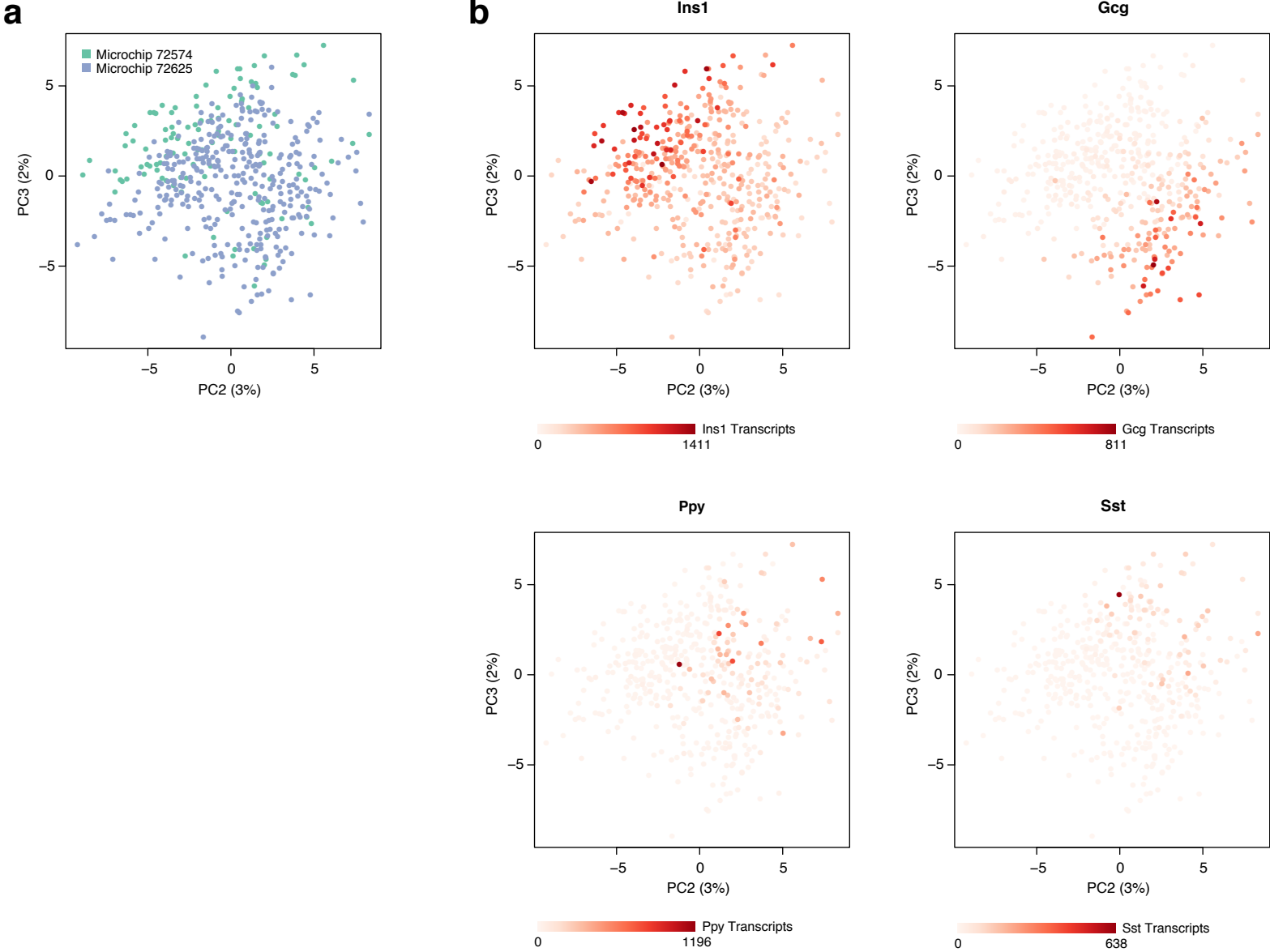

Supplement: Supplementary file 8 — Unsupervised principal component analysis (PCA) for mouse pancreatic islet cells based on the 500 most variable genes. Circles correspond to cells and are colored by (a) microchip (n = 116 for 72574, n = 352 for 72625) and (b) transcript counts of cell type markers (Ins1, Gcg, Ppy, and Sst). (PDF 139 kb) [file 12864_2017_3893_MOESM8_ESM.pdf]

Fig. S9

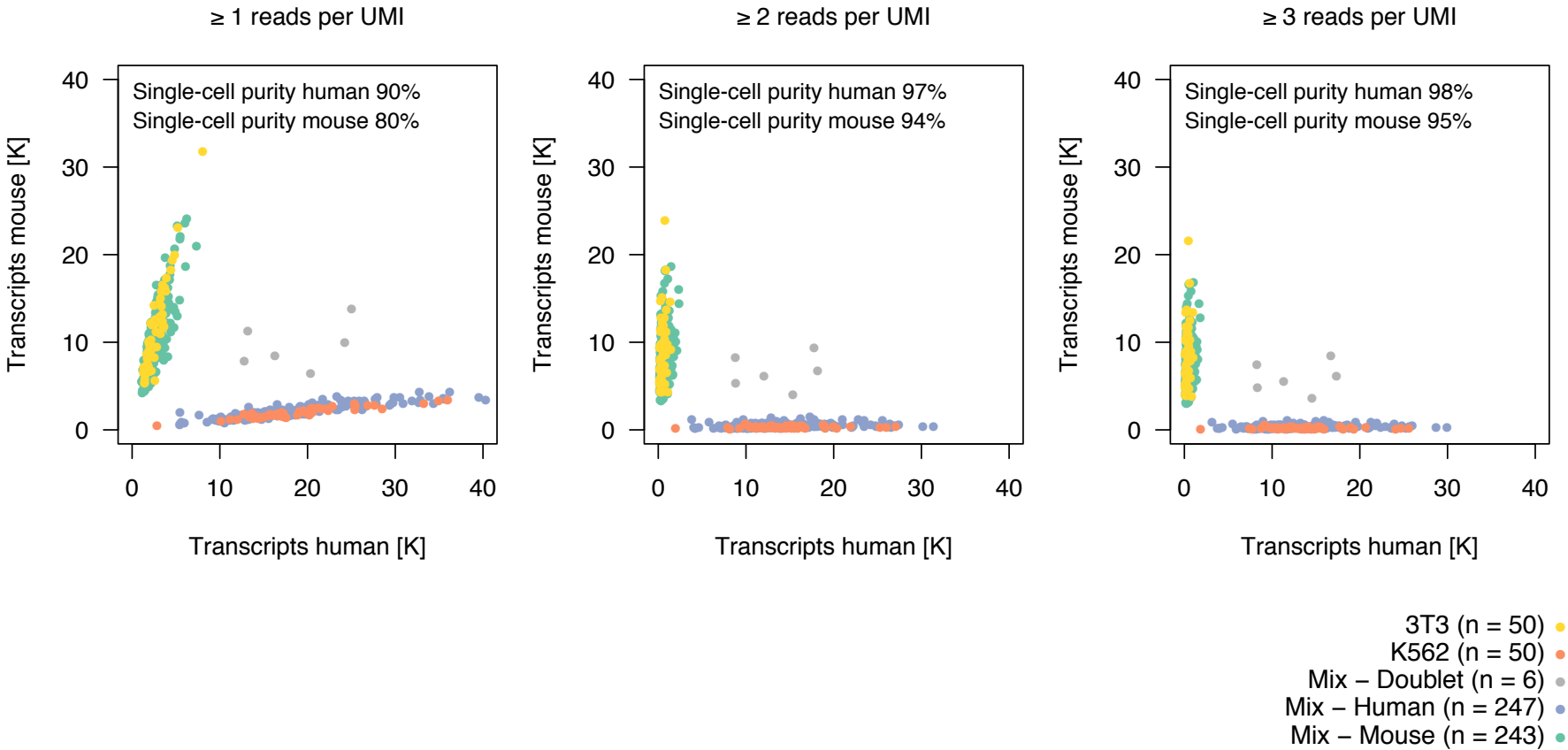

Supplement: Supplementary file 9 — Effect of requiring a minimum number of reads per UMI on single-cell purity. Shown are data for the species-mixing experiment when considering UMIs supported by a minimum of 1, 2 or 3 reads. Single-cell purity estimates are shown in each panel. Otherwise as Fig. 3. (PDF 94 kb) [file 12864_2017_3893_MOESM9_ESM.pdf]
